# Mitochondria inter-organelle relationships in cancer protein aggregation

**DOI:** 10.3389/fcell.2022.1062993

**Published:** 2022-12-19

**Authors:** Ilaria Genovese, Ersilia Fornetti, Giancarlo Ruocco

**Affiliations:** ^1^ Center for Life Nano and Neuro Science, Istituto Italiano di Tecnologia (IIT), Rome, Italy; ^2^ Department of Physics, Sapienza University of Rome, Rome, Italy

**Keywords:** protein aggregation, proteotoxic stress, mitochondria–ER relationship, mitochondria–lysosome relationship, cancer

## Abstract

Mitochondria are physically associated with other organelles, such as ER and lysosomes, forming a complex network that is crucial for cell homeostasis regulation. Inter-organelle relationships are finely regulated by both tether systems, which maintain physical proximity, and by signaling cues that induce the exchange of molecular information to regulate metabolism, Ca^2+^ homeostasis, redox state, nutrient availability, and proteostasis. The coordinated action of the organelles is engaged in the cellular integrated stress response. In any case, pathological conditions alter functional communication and efficient rescue pathway activation, leading to cell distress exacerbation and eventually cell death. Among these detrimental signals, misfolded protein accumulation and aggregation cause major damage to the cells, since defects in protein clearance systems worsen cell toxicity. A cause for protein aggregation is often a defective mitochondrial redox balance, and the ER freshly translated misfolded proteins and/or a deficient lysosome-mediated clearance system. All these features aggravate mitochondrial damage and enhance proteotoxic stress. This review aims to gather the current knowledge about the complex *liaison* between mitochondria, ER, and lysosomes in facing proteotoxic stress and protein aggregation, highlighting both causes and consequences. Particularly, specific focus will be pointed to cancer, a pathology in which inter-organelle relations in protein aggregation have been poorly investigated.

## 1 Introduction

Eukaryotic cellular processes are characterized by the complex relationship between the organized membranous systems composing intracellular organelles. This communication can be conducted through the activation of specific signaling pathways and/or by direct contact or juxtaposition between organelles.

Organelle associations can be both homotypic and heterotypic and involve the endoplasmic reticulum (ER), mitochondria, peroxisomes, lysosomes, Golgi network, plasma membranes, and lipid droplets (Eisenberg-Bord et al., 2016). While homotypic contacts are mostly related to organelle fusion (Stier et al., 1998; Schrader et al., 2000; Eisenberg-Bord et al., 2018), heterotypic contacts consist of juxtapositions in the absence of fusion events and are mediated by molecular determinants and peculiar features, as extensively reviewed elsewhere (Scorrano et al., 2019; Eisenberg-Bord et al., 2016).

In addition to the lack of fusion events, heterotypic contacts are characterized by 1) the presence of tethered proximity (defined approximately in the range between 10 and 80 nm, preferably 30 nm) guided by protein–protein interactions and lipid–protein interactions; 2) a specific function involving the bidirectional transport of ions and molecules; and the exchange of signals involved in organelle homeostasis regulation and enzyme activity regulation; and 3) a defined proteome and lipidome for the maintenance of both contact architecture and function. Indeed, these macromolecules are arranged in a “quasi-synaptic” organization where proteins and lipids can be either resident or migrate from the cytoplasm to the sites where they play specific functions. For instance, ER–mitochondria contacts are described as “raft like” due to their enrichment in sterols and ceramides (Poston et al., 2011; Garofalo et al., 2016).

Furthermore, the protein composition of inter-organelle contacts is extremely specialized due to the presence of diverse protein classes that determine both the function and the architecture of the tethers. Such classes include structural proteins, functional proteins, regulator proteins, and sorter/recruiter proteins (Scorrano et al., 2019).

For its intrinsic dynamic nature, inter-organelle communication is flexible and dependent on cell type, function, and state (Carrasco and Meyer, 2011; Giordano et al., 2013; Idevall-Hagren et al., 2015; Raiborg et al., 2015; Lees et al., 2017). Moreover, given their specific function and tight regulation, organelle contacts might be the result of a fine selection under evolutionary pressure.

In this scenario, mitochondria play a central role in inter-organelle communication and contact establishment. Indeed, being the central hub for ATP production, metabolites production, Ca^2+^ homeostasis regulation, and ROS production, mitochondria play a compelling role in the determination of cell fate (Missiroli et al., 2020b; Genovese et al., 2020; Perrone et al., 2020; Rimessi et al., 2020; Ahumada-Castro et al., 2021; Díaz et al., 2021; Genovese et al., 2021; Modesti et al., 2021; Carreras-Sureda et al., 2022).

Increasing evidence supports the involvement of mitochondrial inter-organelle interactions in several essential cellular responses such as mitophagy, intracellular Ca^2+^ handling, cell proliferation, cell death, and proteostasis (Gelmetti et al., 2017; Marchi et al., 2018; Ali et al., 2021; Fontana and Limonta, 2021; Suárez-Rivero et al., 2022; Zheng et al., 2022). Furthermore, the deregulation of the mentioned processes is oftentimes related to several pathological conditions, from neurodegeneration to inflammation and from cancer to diabetes and cardiac defects (Giorgi et al., 2018; Missiroli et al., 2020a; Liu et al., 2020; Simoes et al., 2020; Morciano et al., 2021; Bouhamida et al., 2022; Patergnani et al., 2022; Tang et al., 2022).

Among all the mitochondrial inter-organelle interactions and communication mechanisms, ER–mitochondria, and mitochondria–lysosomes are the most studied and characterized.

As a matter of fact, the endoplasmic reticulum represents the hub of protein synthesis and participates in the regulation of intracellular Ca^2+^ homeostasis, playing a fundamental role in the regulation of unfolded protein stress responses and cell viability (Oakes and Papa, 2015). On the other hand, lysosomes shield the lytic enzymes necessary for the degradation of damaged organelles, macromolecules, and other intracellular structures, taking part in the complex process named autophagy (Lawrence and Zoncu, 2019).

Mitochondria–ER, and mitochondria–lysosome can communicate by engaging contacts that enable signaling molecule sharing and regulatory complex scaffolding (Díaz et al., 2021). Interestingly, cellular processes involved in pathological mechanisms, such as cancer, are often linked to altered organelle interactions (Boroughs and DeBerardinis, 2015). Nevertheless, the relationship between cancer progression and alteration of inter-organelle interplay is very complex and not lacking in ambiguous aspects (Fouad and Aanei, 2017). This is probably due to the innate propensity of cancer cells to survive and overcome all unfortunate events. For this reason, there is increasing evidence of organelles contact alteration in cancer as a way to respond to stress cues.

In the following section, the molecular determinants, overall composition, and major functions of ER–mitochondria and mitochondria–lysosome interconnections will be described.

Since the great link that connects mitochondria to ER and lysosomes is protein homeostasis (proteostasis), deregulation in protein synthesis and degradation of misfolded or aggregated proteins represent major injuries to cell health. In particular, ER and lysosomes take part in distinct processes; while ER is the prime site where ribosomes synthesize proteins and where chaperones initiate folding processes, lysosomes participate in autophagy/mitophagy when the amount of misfolded protein aggregates crosses the cell tolerance limit. Although mitochondria seem not to participate in proteostasis, evidence supports that defects in mitochondrial protein import increase the rate of protein aggregation in the cytoplasm (Nowicka et al., 2021). Moreover, defects in redox homeostasis and mitochondrial ROS production strongly contribute to protein aggregation and amyloid formation (van Dam and Dansen, 2020).

Seemingly, mitochondria-related protein aggregation could be more easily linked to neurodegenerative conditions, even though it can be generally attributed to age-related diseases, including cancer (Francisco et al., 2020).

This review will focus on the central role of mitochondria and its interactions with ER and lysosomes in the regulation of proteostasis and protein aggregation. This issue, which has been well-characterized and studied in neurodegenerative and aging-related diseases, is still scarcely investigated in a mere cancer setting. Proteostasis regulation in cancer may indeed represent an intriguing, yet not fully elucidated, organelle-coordinated quality control with ambiguous implications for tumor proliferation and survival.

## 2 Mitochondria contacts and communication with the ER and lysosome: cancer implications

### 2.1 ER–mitochondria

Among others, the connections between the ER and mitochondria are the most deeply studied given the multiple implications in disease onset. This aspect is in agreement with the evidence that an impaired organelle-coordinated response is linked to defective adaptation and ultimately to cell dysfunction (López-Crisosto et al., 2015).

The ER–mitochondria distance in mammalian cells, assessed through electron tomography studies, is around 25 nm (Csordás et al., 2006). The same studies also demonstrated that inter-organelle connections take place in specialized domains, named mitochondria–ER-associated membranes (MAMs). The molecular determinants on both involved sides are essential for the regulation of physiological processes such as mitochondrial fission, lipid transport, energy metabolism, Ca^2+^ signaling, autophagy, glucose metabolism, immune signaling, phospholipid synthesis, inflammation, and insulin signaling (Wu et al., 2018; Silva et al., 2020; Vallese et al., 2020).

The very first evidence of ER–mitochondria tethering was found in yeast pertaining to Mmm1, Mdm10, Mdm12, and Mdm34, which together form a complex named ER–mitochondria encounter structures (ERMES) (Kornmann et al., 2009; Kornmann, 2020).

Regarding MAM molecular determinant conservation, although the proteins found enriched at MAMs have no peculiarly conserved amino acid stretches (as would be expected from proteins targeting a specific compartment), cysteine palmitoylation has been reported to be necessary for TMX and calnexin sorting at MAM (Lynes et al., 2012).

Moreover, given the diversity of MAM functions, it is still not clear whether a single or several MAM compartments exist, each providing a function-specific set of proteins. As a matter of fact, the same proteins and enzymes located at MAM can also be found at the mitochondrial or ER surface, as transiently associated upon needs (Schrader et al., 2015).

**FIGURE 1:**
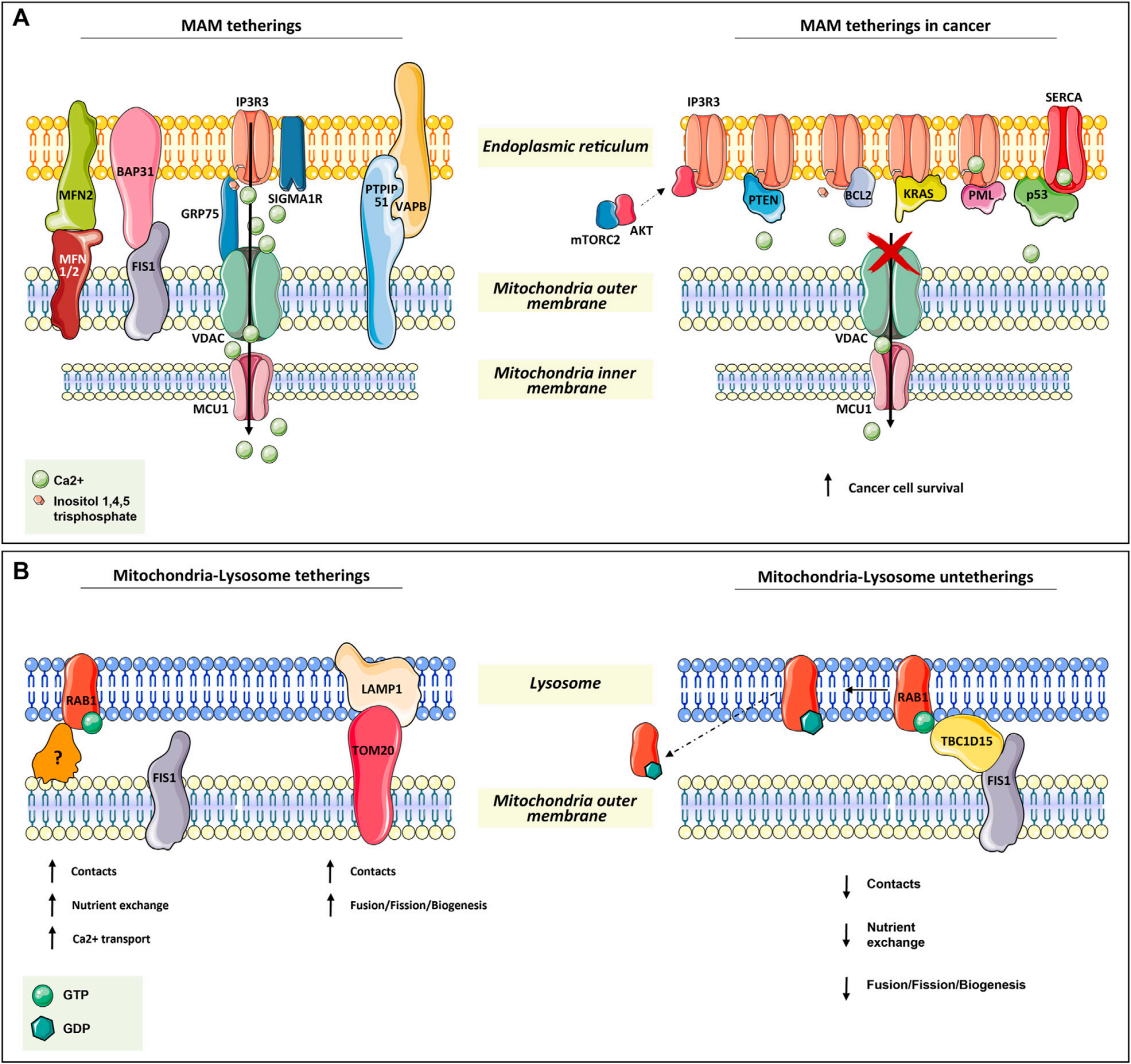
Mitochondria tetherings with ER and lysosomes. **(A)** Mitochondria–ER tethering systems, named mitochondria–ER-associated membranes (MAMs). In the left panel, the architectural and functional systems for mitochondria–ER association are described. Notably, the IP3R3/sigma1R-GRP75-VDAC1-MCU1 axis is both fundamental for membrane proximity and association while being functional for Ca^2+^ homeostasis regulation, as for the other systems in the figure (read Section 2.1). On the right panel, the proteins found transiently located at MAM are depicted. All of them (oncogenes or onco-suppressors) regulated cancer progression through IP3R3 and SERCA, thus acting on mitochondrial Ca^2+^ homeostasis. **(B)** In the left panel, the functional tethering between mitochondria and lysosome for proper nutrient exchange, Ca^2+^ homeostasis, and mitochondria dynamics (fusion, fission, biogenesis). In the right panel, the molecular mechanism involved in mitochondria–lysosome tethering disruption, which is mediated by TBC1D15 on RAB1-GTPase, is depicted (read Section 2.2 for more details).

ER–mitochondria tethering is both structurally and functionally fundamental for proper Ca^2+^ signaling. The more extensively characterized connections include inositol-1,4,5-trisphosphate receptor 3 (IP3R3) on the ER surface, the voltage-dependent anion channel (VDAC), bridged by glucose-regulated protein 75 (GRP75), on the mitochondrial outer membrane, and the MCU complex in the inner mitochondrial membrane. MAMs have been found highly enriched in these proteins since Ca^2+^ is essential for mitochondria-dependent energy metabolism, motility, biogenesis, and apoptosis (Giacomello et al., 2007; Baughman et al., 2011; De Stefani et al., 2011; Rowland and Voeltz, 2012; Chaudhuri et al., 2013) (Figure 1A).

In addition to GRP75, IP3R3 can also be modulated by sigma1R and a promyelocytic leukemia (PML) tumor suppressor (Raturi and Simmen, 2013; Marchi et al., 2014) (Figure 1A).

Another tether involves Mnf2 on the ER surface and Mfn1/2 on the mitochondrial outer membrane. In particular, Mnf2 is considered a *bona fide* constituent of MAM and is involved in proximity, contact sites, and mitochondrial fusion regulation (Schrader et al., 2015; Degechisa et al., 2022) (Figure 1A).

In the same way, VAPB on the ER membrane makes contact with PTPIP51 at the mitochondrial outer membrane, which is an additional physical linker of MAM (Stoica et al., 2014) (Figure 1A).

A tether, which is not only involved in the maintenance of MAM structure but also in a feedback loop for cellular cues, is the ER-resident protein Bap31. In response to sustained mitochondrial Ca^2+^ entry, Bap31 interacts with mitochondria-resident Fis1, recruiting pro-caspase-8 at MAM and promoting its activation to caspase-8 to trigger apoptosis (Iwasawa et al., 2011) (Figure 1A).

Given the importance of these connections in the regulation of both ER–mitochondria physical proximity and their proper functionality, their disruption or dysregulation is expected to have consequences on cell viability. Great effort has been spent in the last few years on the dissection of MAM non-resident proteins or else cytosolic proteins that can transiently localize at MAM to regulate tethers in many pathophysiological contexts. Great attention was given to cancer, neurodegeneration, cardiac diseases, and diabetes. In tumorigenesis, the regulation of ER–mitochondria communication concerns mostly Ca^2+^ transfer. Indeed, the efficiency of ER–mitochondria Ca^2+^ transfer, which greatly affects ATP production and autophagy, is crucial for cancer cell fate (Genovese et al., 2020).

For instance, in several types of tumors at MAM apoptosis-related proteins such as: Bax, Bak, Bcl-2, Bcl-xL (Scorrano et al., 2003; Chami et al., 2004), and other proteins as: PML (Giorgi et al., 2010; Missiroli et al., 2016), p53 (Giorgi et al., 2015, PTEN (Bononi et al., 2013), kRAS (Pierro et al., 2014), and mTORC2/akt (Marchi et al., 2008; Marchi et al., 2012; Betz et al., 2013), were found to be involved in the regulation of Ca^+^-dependent cancer cell survival and propagation. Notably, many of these proteins are well-known tumor suppressors or oncogenes, underlining their complex implications in tumorigenesis (Figure 1A).

For a complete review of MAM-localizing proteins involved in tumorigenesis, refer to the study of Morciano et al. (2018) and Simoes et al. (2020).

### 2.2 Lysosome–mitochondria

The relationship and physical connection between lysosomes and mitochondria are essential for the well-being of both organelles and for cell viability. Indeed, there is evidence that the malfunctioning of mitochondrial proteins or the inhibition of the electron transport chain also impairs lysosomal activities (Demers-Lamarche et al., 2016).

The simplest connection between lysosomes and mitochondria takes place during autophagy/mitophagy, when either the entire organelle or mitochondria-derived vesicles fuse directly with lysosomes to be degraded (McLelland et al., 2014). Interestingly, the coordinated dysfunction of both organelles has been reported as associated with many pathological conditions, including cancer (Osellame et al., 2013; Baixauli et al., 2015; de la Mata et al., 2015; Norambuena et al., 2018).

Another piece of evidence demonstrating the importance of the lysosome–mitochondria relationship consists in the role of the transcription factor EB (TFEB), which promotes both lysosomal and mitochondrial biogenesis and the expression of oxidative phosphorylation enzymes (Mansueto et al., 2017). Moreover, the inhibition of lysosomal acidification affects the mitochondrial maximal oxygen consumption rate (Monteleon et al., 2018).

Nonetheless, the contacts between mitochondria and lysosomes are not only aimed at mitophagy but also directed to non-degradative functions, such as calcium and iron transfer, lipid homeostasis, and mitochondrial dynamics (biogenesis, fusion, and fission) (Wong et al., 2018; Wong et al., 2019). In confirmation of this, the knock-out of five autophagy receptors has no effect on mitochondria–lysosome contacts (Chen et al., 2018).

The main player in mitochondria–lysosome tethering is the GTPase Rab7, a well-known regulator of late endosome/lysosome dynamics. Membrane-bound active Rab-GTP is able to recruit effector proteins to the lysosome in order to promote lysosome fusion, transport, and contact with other organelles, such as mitochondria (Wong et al., 2019). Experimental evidence has demonstrated that the overexpression of a non-hydrolyzable and constitutively active Rab7 GTP increases the number and duration of mitochondria–lysosomal contacts (Wong et al., 2018). Conversely, the contact disruption is mediated by the GTPase-activating protein (GAP) TBC1D15, which is recruited at the mitochondrial surface by the mitochondrial outer membrane resident protein Fis1 (Zhang et al., 2005; Peralta et al., 2010; Onoue et al., 2013). The efficacy of TBC1D15 in disrupting this contact is strictly dependent on its mitochondrial localization. In fact, the overexpression of the Fis1 (LA) mutant, which is unable to recruit TBC1D15, and Fis1 knock-out result in an increment of mitochondria–lysosome contact number and duration (Wong et al., 2018) (Figure 1B).

In addition, immunofluorescence and 3D SIM imaging experiments have revealed that mitochondrial Tom20 and lysosomal Lamp1 make contact (Wong et al., 2018). This is also supported by the evidence that the majority of mitochondrial fission events are linked to Lamp1-positive vesicles, which further strengthens the indication of lysosome’s role in mitochondrial dynamics (biogenesis, fission, and fusion) (Wong et al., 2018) (Figure 1B).

Thus, lysosomes are crucial for mitochondrial wellness because they are involved in energy homeostasis, transcriptional activation, and cell growth. This has been demonstrated in various pathological settings, especially in cancer, where these organelle relationships are involved in tissue priming for angiogenesis and metastasis dissemination (Davidson and Vander Heiden, 2017).

Moreover, a research study demonstrated that lysosomal V-ATPase, known for being involved in apoptosis induction, hinders lipid metabolism in cancer through a dysfunctional relationship with mitochondria (Bartel et al., 2017). Specifically, alterations in lysosome functions affect lipid composition and localization, consequently triggering mitochondrial fission, a shift to glutamate metabolism (typical of cancer cell metabolism), loss of membrane potential, and reduction of oxidative capacity. Altogether, these events lead to an increase in ROS levels and apoptosis induction (Bartel et al., 2019).

## 3 Role of mitochondria in protein aggregation

In order to maintain correct proteostasis, cells exploit three molecular machineries that often act in a linked and sequential manner: 1) molecular chaperones for protein folding/refolding; 2) the ubiquitin proteasome system; and 3) the autophagy processes for eliminating the misfolded proteins and aggregates. Whenever one of these systems fails in the elimination or rescue of misfolded proteins, protein aggregates tend to accumulate, inducing cytotoxicity.

During the folding process, a crucial step for proteins to reach their final tertiary conformation is cysteine oxidation for disulfide bridge formation. For this reason, protein aggregation can be influenced and regulated by the cellular redox state (Holmström and Finkel, 2014). In some cases, upon alteration of the redox state, conformational changes lead to the protein switching to a partially unfolded state that might translate into aggregate formation. Many proteins also contain intrinsically disordered regions, predicted to fold upon protein–protein interactions (Malagrinò et al., 2022), and conditionally disordered regions, able to promote the transition from disordered to ordered states depending on reduction or oxidation of their residues (Erdős et al., 2019). Interestingly, protein folding is determined by the oxidation/reduction of cysteines and methionines, but at the same time, misfolded proteins are more sensitive to oxidation (Dukan et al., 2000).

Oxidation mechanisms are not only detrimental but can also be regulatory for protein functions. For instance, in some cases, amyloid fibril formation is not permanent because cysteines are reversibly oxidized (Audas et al., 2016). An example of this mechanism is the regulation of the p16INK4A tumor suppressor through the oxidation of its only cysteine that causes a rapid yet reversible formation of disulfide bridge-dependent homodimer β-amyloid fibrillation. Interestingly, this mechanism of protein inactivation allows for its cyclic regulation through the cell cycle phases (Göbl et al., 2020).

Often, cysteine and methionine oxidation is involved in intermolecular disulfide bridge formation and permanent alteration of protein structure that results in aggregate accumulation. In turn, the aggregation is strictly related to the increase in oxidative stress and ROS production that cause proteinopathies in many pathological conditions from cancer to neurodegeneration (van Dam and Dansen, 2020).

**FIGURE 2:**
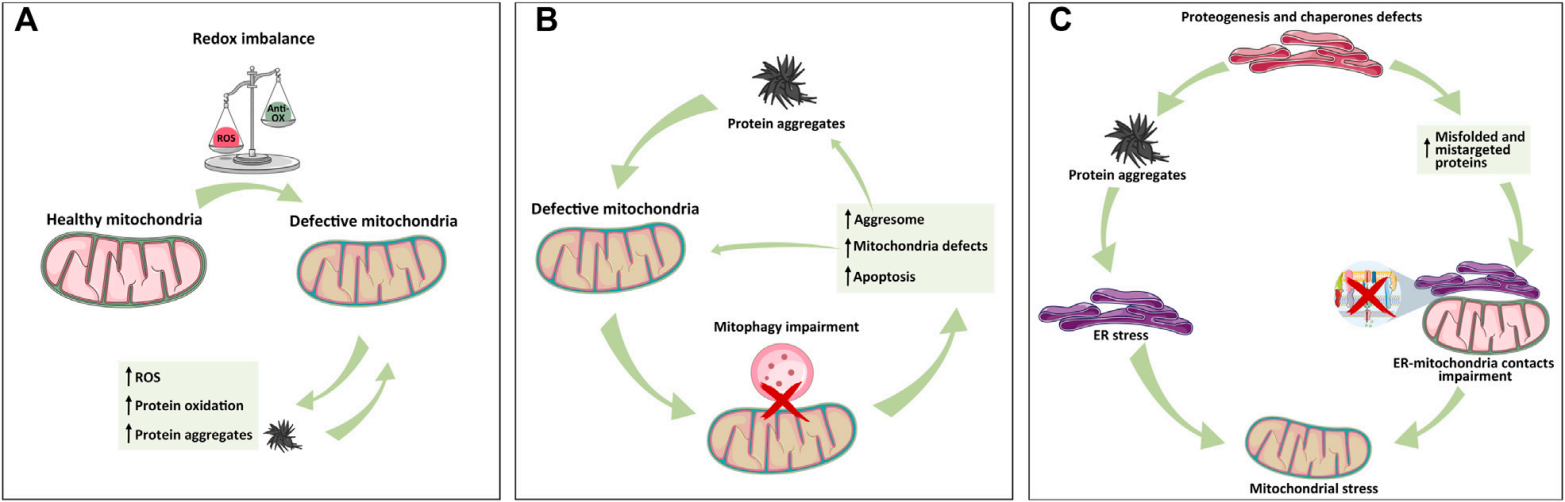
Mitochondrial relationship with ER and lysosome in protein aggregate formation. **(A)** Mitochondria-dependent redox equilibrium can be altered by endogenous or extracellular cues, leading to the accumulation of ROS and protein aggregates that, in turn, exacerbate the vicious circle of mitochondria impairment and protein aggregation. **(B)** Following protein aggregate accumulation and mitochondrial impairment, lysosome–mitochondria communication for mitophagy is impaired, leading to mitochondria stress, activation of aggresome, protein aggregates formation, and apoptosis activation whether mito/autophagy cannot rescue the cell. **(C)** ER is the hub of protein synthesis. Defects in proteogenesis, chaperone activity, and signal recognition particles (SRPs) increase the accumulation of unfolded protein and thus aggregates, triggering ER stress, and MAM alteration, all of which cause mitochondrial defects. As depicted in panel A, the exacerbation of protein aggregates formation leads to organelle failure. The simplification of these inter-organelles relations into these three panels has the only aim of highlighting the individual contributions in a more complex and intertwined setting.

Mitochondrial dysfunctions caused by an impairment in Ca^2+^ homeostasis, energy production, or mitophagy represent the major cause of ROS level deregulation. Consequently, mitochondria-dependent alteration of the redox environment is closely associated with protein oxidation and thus aggregation. As mentioned, the accumulation of protein aggregates further exacerbates mitochondrial dysfunctions, leading to more metabolic stress, ROS production, and finally mitochondria-dependent apoptosis induction (Hashimoto et al., 2003; Schubert et al., 1995; Curtain et al., 2001) (Figure 2A).

Interestingly, mitochondrial proteins themselves can suffer from aggregation, mostly because of errors in the coordinated communication between mitochondria–ER–cytoplasm. Indeed, mitochondrial inner membrane-integrated proteins produced in the cytoplasm have to cross two membrane layers and pass through extremely narrow pores that exclude globular folded proteins from translocation. For this reason, the mitochondrial pre-proteins are inserted mainly in unfolded forms, exposing mitochondria to non-native proteins through constant contact. These pre-proteins, after the cleavage of the N-terminal signal sequence, are folded by the mitochondrial folding machinery through a finely tuned series of events. Defects occurring at any of these stages can easily lead to the accumulation of misfolded proteins inside the mitochondria, exacerbating its failure and proteotoxic stress (Mokranjac and Neupert, 2005; Neupert and Herrmann, 2007; Alder et al., 2008; Mokranjac and Neupert, 2009; Mokranjac, 2020; Guo et al., 2021).

In addition to problems in the coordinated action of different cellular compartments in protein translocation and folding, mitochondrial intrinsic characteristics can represent a challenge for protein folding and potential aggregation. A recent study reported that intra-mitochondrial temperature (50°C) is higher than that in other organelles and sub-compartments, complicating the folding challenge, especially for mitochondrial metastable proteins (Chrétien et al., 2018).

In this landscape, alteration in proteostasis caused by mitochondria can be easily linked to aging-related pathologies, in which cells suffer from altered redox state maintenance and decay in rescue systems. This mechanism, poorly investigated in other types of diseases, can be applied to all cellular contexts where mitochondria-altered metabolism causes an imbalance in the redox state, leading to toxic protein aggregation.

Notably, ROS-dependent protein aggregation is not merely detrimental, as on the one hand, ROS enhances protein aggregation, and on the other hand, ROS-induced aggregation can also act as a cellular strategy to enhance the clearance of toxic protein-soluble aggregates (van Dam and Dansen, 2020).

Certainly, the connection between mitochondria-dependent ROS production, redox signaling, and proteostasis is a complex relationship where causes and consequences are difficult to distinguish. Small variations in the cellular redox state or protein aggregation state can be sufficient to turn a regulatory and potentially protective mechanism into a toxic and irreversible situation.

Cancer can be partly considered an aging disorder, where cells accumulate fatal defects and mutations because of mitochondrial homeostasis alteration. Indeed, cancer cells also suffer from proteostasis defects.

Supporting the role of mitochondria both as player and as victim of oxidative stress-induced protein aggregate accumulation, a recent research study has highlighted that protein aggregation in luminal breast cancer mostly involves mitochondrial proteins and that it is induced by the anti-estrogen treatment 4OH-tamoxifen, known to generate oxidative stress (Ahmed et al., 2021; Direito et al., 2021).

Regarding this topic, a study showed that the accumulation of proteins in mitochondrial intermembrane space activates a mitochondrial-specific UPR (mtUPR) in breast cancer cell lines. As a consequence of protein accumulation, ROS overproduction triggers estrogen receptor activity that further regulates the transcription of mitochondrial proteases, biogenesis factors, and proteasome activation. This suggests that estrogen receptor-mediated mtUPR activation has cytoprotective effects from ROS overproduction (Papa and Germain, 2011).

Additionally, proteotoxic stress influences mitochondria through a crucial transcriptional factor, heat shock factor 1 (HSF1). HSF1 is essential for mitochondrial chaperones and protease transcription, especially during mtUPR, thus acting as a mitochondrial function guardian upon impaired protein homeostasis (Katiyar et al., 2020). For instance, mtUPR inhibitors disrupt the interaction and cooperation of mitochondrial chaperones and proteases, triggering metabolic stress and arresting cell proliferation, suggesting that mtUPR is essential for tumorigenesis (Kumar et al., 2022).

As a matter of fact, HSF1 decrease promotes apoptosis after protein amyloid accumulation in Alzheimer’s disease; therefore, amyloidogenesis can be considered a checkpoint for uncontrolled growth and survival (Tang et al., 2020).

Moreover, HSF1 antagonizes AMP-activated protein kinases, driving a lipogenic phenotype that supports melanoma growth (Su et al., 2019). Indeed, HSF1 activation promotes tumoral growth and pre-malignant cell development by metabolic stimulation, specifically by lipid biosynthesis. Evidence demonstrates that HSF1 inactivation decreases hepatocellular carcinoma progression by regulating insulin sensitivity, lipid metabolism, and energy homeostasis (Jin et al., 2011).

Moreover, it has been demonstrated that the RAS-MEK pathway is fundamental for HSF1 functions since the MEK–HSF1 interaction regulates proteostasis surveillance and amyloidogenesis suppression. When MEK is blocked, HSF1 is consequently inactive, provoking proteotoxic stress accumulation due to aggregation and amyloidogenesis. Tumor cells are susceptible to proteomic alteration and aggregate accumulation; as a matter of fact, amyloidogenesis is tumor suppressive. Even though proteomic instability is one of the features of the malignant state, the promotion of amyloid formation and accumulation may be a viable therapeutic strategy (Tang et al., 2015).

These studies indicate that tumors engage in mitochondrial chaperone transcription and metabolism rewiring in order to survive proteotoxic stress.

In conclusion, mitochondria-dependent cellular redox state, mitochondrial-dependent survival strategies, and protein aggregation are tightly connected, either to support a protein regulatory function or to activate protein aggregate clearance, with both physiological and pathological significance. For instance, mitochondrial damage in rat breast cancer cells leads to the accumulation of p53 aggregates that are resistant to lysosome degradation and association with autophagic receptors (NDP52 and TAX1BP1), enhancing cancer cell growth (Biel et al., 2020). The targeting of post-misfolding oxidation events or mitochondrial-dependent survival pathways might open new therapeutic strategies for cancer treatment. Regarding p53 aggregation, thiol-reactive compounds have been shown to induce the refolding and reactivation of mutant p53. This evidence could pave the way toward new convergent therapeutic strategies for proteinopathies and cancer (Zhang et al., 2018).

## 4 Protein aggregation’s role in mitochondrial interactions with ER and lysosomes: causes and consequences

As described in the previous paragraph, mitochondria produce ROS that can be either functional or detrimental to protein aggregate clearance or accumulation. Mitochondria do not act alone in these mechanisms, since they play a key role in proteostasis through their connections with other organelles.

For example, mitochondria are physically proximal to the ER, where surface-associated ribosomes produce polypeptide chains. Sometimes, mitochondria encounter mistargeted proteins from the ER, especially when there is a lack of the availability of signal recognition particles (SRPs). The accumulation of these proteins can trigger mitochondrial proteotoxic stress (Ali et al., 2021) (Figure 2C).

Additionally, ER stress causes superoxide radical production through the activation of NADPH oxidases (NOX) and Ca^2+^ release that causes electron leakage from mitochondria (Santos et al., 2009).

Thus, ROS can be both the cause and consequence of mitochondrial and ER stress, exacerbating the vicious circle of stress accumulation and proteostasis imbalance (Malhotra and Kaufman, 2007) (Figure 2C).

An interesting study highlights that protein aggregation in yeast is induced by acute stress. Nascent proteins are key players in aggregation initiation since freshly translated polypeptide chains have to overcome prolonged periods under stress conditions and environmental changes. In the cytosol, nascent misfolded proteins act cooperatively with other misfolded proteins in maintaining and promoting the misfolded state. On the other hand, the stabilization of a nascent protein is supported by its interaction with other properly folded proteins. These opposing stabilizing effects on native versus misfolded states can be pictured by imaging the protein aggregation-forming phase separation induced by freshly translated polypeptides near the translation sites. In the same research regarding yeast protein aggregation, a coordinated role of mitochondria and ER in the formation of protein aggregates was found. This evidence reflects the predisposition of protein aggregates to bind the ER and mitochondrial surface proteins without overlapping with tethering proteins. This evidence possibly suggests the existence of an additional bridging system between these organelles (Zhou et al., 2014).

Another study demonstrates that misfolded proteins formed at the ER surface associate and are partly transported into the mitochondria, possibly leading to mitochondrial dysfunction. Furthermore, the blockage of the ER–mitochondria tethers, but not the mitochondrial sorting and assembly machinery (SAM) or mitochondrial surveillance pathway regulators, dampens mitochondrial sequestration of ER-derived misfolded proteins. The study also demonstrated that ER-associated mitochondrial sequestration (ERAMS) has a role in the management of protein aggregates and proteostasis in general and in pathologically related mitochondrial dysfunction (Cortés Sanchón et al., 2021). Notably, there is more than one piece of evidence about the accumulation of misfolded cytosolic proteins at ER–mitochondria proximity regions, which are later translocated inside the mitochondria (Zhou et al., 2014; Ruan et al., 2017; Li et al., 2019; Shcherbakov et al., 2019; Ruan et al., 2020).

As described in the previous paragraph, lysosome–mitochondria *liaison* is focused on the regulation of mitochondrial dynamics (mitophagy/biogenesis/fission/fusion) and metabolism. These mechanisms are powered by the transfer of metabolites (amino acids and lipids) from the lysosome to mitochondria to aid the Krebs cycle and lysosome-mediated mitochondrial degradation through mitophagy (Elbaz-Alon et al., 2014; Hönscher et al., 2014; González Montoro et al., 2018; Kumar et al., 2018).

The autophagy master regulator mTORC1 has a central role in the anti-aging defense mechanism since it collaborates in processes such as protein synthesis reduction, metabolic reprogramming, and stress response, supporting biomass production and mitochondrial respiration over glycolysis (Zhao et al., 2015; Lawrence and Zoncu, 2019).

In a cancer setting, the autophagy–lysosome system is crucial for the survival and dissemination of KRAS-mutated adenocarcinomas, which are characterized by the ability to grow in nutrient- and oxygen-deficient environments (Guo et al., 2011; Yang et al., 2011; Commisso et al., 2013; Mancias et al., 2014; Perera and Bardeesy, 2015; Perera et al., 2015; Davidson et al., 2017).

The scavenging and recycling of intracellular components converge on the lysosome. Indeed, lysosome-mediated degradation of organized macrostructures such as mitochondria and peroxisomes has an essential role in nutrient supply in cancer growth and adaptation. This could point toward the development of new therapeutic strategies exploiting lysosome pH disruption in combination with chemotherapeutic treatment (McAfee et al., 2012; Piao and Amaravadi, 2016; Rebecca et al., 2017).

Regarding protein aggregate clearance, cells take advantage of another mechanism known as the “aggresome.” The aggresome recruits motor proteins that translocate aggregates to chaperones and/or proteasomes for their destruction. The hindering of the aggresome pathway facilitates the accumulation of aggregated proteins and subsequent activation of apoptosis and mito/autophagy in cancer cells (Rodriguez-Gonzalez et al., 2008). The lack of protein aggregate removal activates autophagy, which degrades mitochondria in order to correct their dysfunction. As a consequence, aggresomes are engulfed by the autophagosome and fused to the lysosome to enable the degradation of the proteins (Rodriguez-Gonzalez et al., 2008) (Figure 2B).

Recent evidence highlights that aggresome and autophagy pathways are intertwined. Parkin-mediated K63-linked polyubiquitination connects misfolded proteins to the motor protein complex through histone deacetylase 6 (HDAC6) to finalize aggresome formation and degradation by autophagosomes (Olzmann and Chin, 2008). This mechanism helps eliminate the accumulated misfolded polyubiquitinated proteins observed in proteinopathies. Therefore, HDAC6-mediated transport of polyubiquitinated misfolded proteins into aggresomes helps the final degradation through autophagy (Garcia-Mata et al., 2002; Boyault et al., 2007). Since aggresome formation and autophagy are linked to cancer, the synergistic targeting of these two mechanisms can represent a successful therapeutic strategy.

As previously stated, proteinopathies are often associated with neurodegenerative and aging-related diseases (Stefani and Dobson, 2003); nevertheless, p53, the most well-known tumor suppressor, was found to form amyloid fibrils and undergo aggregation in diverse types of cancer (Silva et al., 2013), indicating that cancer might also be considered a protein aggregation disease. Usually, even though the amino acid sequences of amyloid-forming and aggregation-prone proteins are very diverse, the process of aggregation is conserved and starts from the state of insoluble but highly organized cross-β spine. Then, aggregate accumulation depends on protein concentration, interaction with other partners, the overall environment, and defects in misfolded protein clearance (Aguzzi and O’Connor, 2010).

Regarding p53, many research studies have demonstrated that its transactivation and DNA-binding and tetramerization domains have the potential to misfold and form fibrillary aggregates *in vitro* (Ishimaru et al., 2003; Higashimoto et al., 2006; Rigacci et al., 2008). Moreover, the phenomenon was also observed in different tumor cell lines and cancer types (Xu et al., 2011; Silva et al., 2013).

Furthermore, p53 aggregates can sequestrate the native form of p53, inactivating its pro-apoptotic functions, and this event may be a key modulator of cancer cell propensity to proliferate, spread, and resist death stimuli.

Although both wild-type and mutant p53 can form aggregates, very little is known about the causes triggering p53 aggregation. A possible cause could be the accumulation of misfolded p53, p53 imbalanced turnover, or ROS accumulation, mitochondrial dysfunction, and metabolic stress (Yang-Hartwich et al., 2015).

A recent study showed that mutated p53 can be degraded *via* the proteasome due to mitochondria-targeting aggregation-induced emission (AIE) materials. AIE elicits mitochondrial stress and ROS overproduction, rescuing cancer cell lines from mutated p53 (Wang et al., 2022).

Moreover, other evidence supports the complex role of p53 in antagonizing cancer cell growth. Indeed, p53, by interacting with WWOX and TIAF1, suppresses cancer cell proliferation and migration. However, p53 may also antagonize the WWOX tumor suppressor by dampening WWOX-dependent inhibition of cancer-related inflammatory responses. Interestingly, the p53/WWOX cancer mice display an increased protein aggregation, β amyloid, and tau tangle formation in the brain and lungs, highlighting an overlapping mechanism between cancer, inflammation, and neurodegeneration (Chou et al., 2019).

The tumor suppressor PTEN, which shares functions very similar to p53, is often deficient in cancer and mutated in neurological disorders (PTENopathies) (Cupolillo et al., 2016; Igarashi et al., 2018; Yehia et al., 2019). PTEN aggregation propensity has been studied with computational methods, both in wild-type and mutants. This study revealed that wild-type PTEN can aggregate under physiological conditions and that several mutants increased the aggregation propensity (Palumbo et al., 2020).

Intriguingly, both p53 and PTEN are onco-suppressors reported as transiently associated at MAMs, where they participate in the regulation of mitochondrial Ca^2+^-mediated cell survival, underlining the tight relation between the ER, mitochondria, and lysosomes in the regulation of cell survival, particularly in tumorigenesis (see Section 2.1).

On the other hand, protein aggregation may also occur in oncogene-related pathways. As for PMEL (premelanosome protein), amyloid extracellular aggregates were found in metastatic melanoma together with other proteins that aid amyloid maturation into fibrils. Beta-secretase 2 (BACE2) is involved in the maturation of these aggregates and the induction of yes-associated protein (YAP) activity. Melanoma cancer aggressiveness is associated with YAP-dependent transcription of pro-proliferative genes. Moreover, PMEL fibrils are sufficient to induce transduction that activates YAP signaling. Indeed, the inhibition of BACE2 decreased both extracellular aggregate formation and YAP-related cell proliferation (Matafora et al., 2020).

## 5 Mitochondria-dependent protein aggregation at the crossroad between neurodegeneration and cancer: Possible bivalent therapeutic strategies

Proteinopathies have been intensively studied in the etiology of neurodegenerative diseases (NDs), from Alzheimer’s disease (AD) to Parkinson’s disease (PD) and amyotrophic lateral sclerosis (ALS). Herein, the imbalance in protein homeostasis causes the extracellular or intracellular accumulation of fibrils or insoluble protein aggregates, leading to neuronal defects, degeneration, and eventually programmed cell death. Placing their biological strategies on the opposite side of ND, cancer cells activate a series of molecular pathways to survive and proliferate at any cost.

It has been found that most upregulated genes in ND encode for disordered proteins, while the more frequently upregulated genes in prostate, colorectal, and lung cancer are less prone to form aggregates (Klus et al., 2015).

Although protein structural disorder is more prevalent in ND, the contribution of intrinsic structural disorder or generally protein aggregation cannot be excluded from tumorigenesis (see the previous paragraph). As a matter of fact, the looseness of unfolded proteins can possibly trigger cell death signaling (Gsponer et al., 2008). Indeed, the aberrant expression of intrinsically disordered proteins affects the oncogene Bcl-2 interaction network, leading to a switch in its antiapoptotic function (Rautureau et al., 2010).

Moreover, some cancers are associated with protein-insoluble deposits that influence both tumor survival and metastasis dissemination (de Oliveira et al., 2015). For instance, the co-aggregation of toxic amyloid β peptide and TGF-β-induced antiapoptotic factor (TIAF1) is a strong indication of metastasis development (Chang et al., 2012; Hong et al., 2013). In some cases, aggregate-prone mutated proteins with an important role in ND have been shown to be associated with tumorigenesis. For instance, familiar ALS-associated mutated SOD1 has been demonstrated to boost estrogen-responsive gene expression in breast cancer (Stathopulos et al., 2003; Rao et al., 2008).

A possible connection between cancer and ND is highlighted in a recent study demonstrating that the expression of macrotubule-associated protein (Tau/MAPT), aggregated when excessively phosphorylated in AD, correlates to glioma growth arrest. Furthermore, Tau plays a role in the taxane resistance mechanism of breast, ovarian, and gastric cancers, since both taxanes and Tau share the same binding site for tubulin (Gargini et al., 2019). Not only does Tau slow down the evolution of tumors, especially gliomas, but also pathological Tau can be activated in the brain by glioblastomas secreting CD44, suggesting another connection between cancer and neurodegeneration (Lim et al., 2018).

Apparently, higher Tau expression correlates to a better prognosis for specific types of cancer, while in tauopathies, alterations in Tau expression, modification, or splicing might facilitate neurodegenerative progression. It can be speculated that Tau expression and function might be deregulated during both cancer progression and AD, highlighting a common field of action for this protein. Nonetheless, the literature and experimental evidence agree that the mechanism for Tau inhibition has two facets: post-translational modification as a trigger for tauopathies and AD and changes in Tau expression for gliomas.

Since protein aggregation in proteinopathies and ND often derives from mutations in key proteins for brain functions, evidence shows that it might also be important in a seemingly different pathology such as cancer. Nonetheless, the major common feature in ND and cancer is the direct or indirect role of mitochondria in stress response regulation.

Indeed, mitochondrial damage, in addition to its interplay with other organelles, specifically ER and lysosomes, alters the quality control in ND. Even though the causative effects are ambiguous and the triggering events are hardly traceable, the connection between disease progression, protein aggregate accumulation, and mitochondrial damage is crystal-clear.

In ND, as in cancer, the dysfunction of the coordinated action of mitochondria, ER, and lysosomes leads to impairments in mitochondrial dynamics, Ca^2+^ homeostasis, and metabolism, ultimately inducing protein aggregation. Several examples exist, as in the case of the mutant amyloid precursor protein (APP) that alters mitochondrial dynamics, functionality, and Ca^2+^ signaling (Wang et al., 2008). Other examples are mitophagy, which is often deregulated in PD, and mutant huntingtin, which is found to be associated with the mitochondrial outer membrane influencing mitochondrial permeability transition pore opening (mPTP). Also, the ALS-related SOD1 mutation (G93A) is associated with mitochondrial respiratory dysfunction and severe mitochondria-induced oxidative stress of proteins and lipids (for a complete review on the mitochondria role in ND, refer to the studies of Jishi and Qi (2022) and Ali et al. (2021).

In addition to the aforementioned mechanisms, estrogen receptor-mediated mtUPR might represent a connection between ND and cancer. MtUPR is a cytoprotective strategy engaged by cancer cells to survive misfolded protein accumulation inside the mitochondria. It is triggered by the overproduction of ROS and culminates with proteasome activation (Papa and Germain, 2011).

Indeed, estrogen receptor-mediated mtUPR has a role not only in breast cancer cells but also in ALS. A research study demonstrated that estrogen receptor signaling helps female mice better cope with G93A-SOD1 aggregates inside the mitochondrial intermembrane space than SOD1-mutated male individuals. Indeed, in the absence of estrogen receptors, G93A-SOD1 aggregates are unable to activate the cytoprotective pathways, suggesting a strong correlation between the estrogen receptor-mediated mtUPR and the stress response to protein aggregates both in cancer and ALS (Riar et al., 2017). Looking at mitochondrial protein aggregation as a possible overlap between ND and cancer, the possibility of common therapeutic strategies cannot be excluded. Indeed, drugs used in the ND treatment, such as thioridazine (Sachlos et al., 2012), have been shown to exhibit anti-tumor effects, whilst anti-tumor drugs, such as cyclin-dependent kinase inhibitors (Park et al., 1998) and mithramycin (Sleiman et al., 2011), have shown neuroprotective effects.

Moreover, a recent screening study involving an oligopyridylamide compound library, known for inhibiting AD-related amyloid formation, identified ADH-6 as the lead molecule that abrogates the self-assembly of the p53 DNA-binding domain aggregation-nucleating mutant. This dissociation effect on p53 aggregates has been observed in human cancer cells, where cell cycle arrest and apoptosis were restored upon the treatment (Palanikumar et al., 2021). This experimental evidence represents a huge accomplishment in the successful application of an established amyloid inhibitor as an anti-cancer agent. Altogether, these findings reinforce the existence of a link between cancer and ND, where mitochondria and proteostasis have a major yet ambiguous role, suggesting that novel therapies should point toward converging molecular pathways.

Nonetheless, it is worth remembering that cancer cells are subdued to a much higher mutation load than ND. For this reason, in addition to the promotion of tumor suppressor aggregation (see the previous paragraph), tumors develop a survival strategy to rely more on chaperones, proteasome activity, and autophagy to get rid of the mutant misfolded protein accumulation. Indeed, a recently published paper has demonstrated that the transcription of systems regulating protein aggregation in neurons is weaker than that of other cell and tissue types. This evidence strongly underlies the notion that neurons’ vulnerability to protein misfolding is much higher (Kundra et al., 2020).

From a therapeutic point of view, drugs that modulate oxidative stress, such as melatonin, and UPR, such as PERK or GRP78 inhibitors, can be exploited to enhance cancer cell apoptosis (Obacz et al., 2017; Ojha and Amaravadi, 2017; Xu et al., 2018; Mahameed et al., 2020; Pavlović and Heindryckx, 2022; Xu et al., 2022), while, on the other hand, the inhibition of UPR induction and ROS generation, using antidepressant or antioxidant, can rescue the neurodegenerative phenotypes (Hetz and Mollereau, 2014; Scheper and Hoozemans, 2015; Halliday et al., 2017; Remondelli and Renna, 2017; Hughes and Mallucci, 2019; Srinivasan et al., 2020; Ajoolabady et al., 2022). An interesting study about therapeutic strategy for acute myeloid leukemia (AML) highlighted that a treatment that combines a retinoic acid differentiating agent, an ER stressor tunicamycin, and the oxidative stress inducer arsenic trioxide successfully hinders cancer cell proliferation, eventually inducing apoptosis (Masciarelli et al., 2019). This evidence reinforces the tight relation of ER stress, autophagy, and mitochondrial-dependent oxidative stress to protein aggregation in cancer.

## 6 Concluding remarks

Mitochondria coordinate intricate yet fundamental relations with other organelles in order to maintain cell physiology. Among these relationships, the interactions with the ER and lysosomes are the most important for cell metabolism and survival. Protein aggregation stands at a crossroads in these signaling mechanisms, being either beneficial or detrimental, both the cause and consequence of mitochondrial, ER, and lysosome failure.

The molecular strategy by which mitochondria respond to stress signals often begins with an antioxidant response to face increased ROS production. Oxidative stress not only hampers the proper functions of both ER and lysosomes but also harms macromolecules, especially proteins, causing their aggregation and misfolding.

Among the cellular strategies to overcome stress-related misfolded protein accumulation, in addition to the ER unfolded protein response (UPR), mitochondria engage a mechanism of stress response (mtUPR) following defects in oxidative phosphorylation, ATP depletion, dissipation of mitochondrial membrane potential, pathogen infections, proteostatic stress, and mtDNA alterations (Yoneda et al., 2004; Haynes and Ron, 2010). Just as for ER-related UPR, the accumulation of mitochondrial damage induces the activation of transcription factors that enhance the expression of genes coding for mitochondrial chaperones, proteases, and antioxidant enzymes to minimize misfolded protein accumulation in the organelle, and mitochondria dynamics-related genes (Aldridge et al., 2007; Nargund et al., 2012; Tian et al., 2016; Yi et al., 2018). ATF5 is an mtUPR master regulator, and its expression is influenced by CHOP and ATF4 (Quirós et al., 2017), well-known master regulators of ER-related UPR. This suggests that UPR is part of a more complex integrated stress response that combines ER and mitochondrial signaling pathways.

Recently, much attention has been given to mtUPR’s role in pathological settings such as neuronal disease and cancer, where it is considered helpful for cellular homeostasis after proteotoxic stress accumulation. Indeed, mtUPR is a very conserved pathway in cancer and is often activated in response to mitochondrial stress to sustain cancer cell growth, metabolism, and survival (Papa and Germain, 2011; Jenkins et al., 2021; Inigo and Chandra, 2022). In addition, mtUPR-dependent gene expression activation is a strategy for the selective adaptation of cancer cells during the stress integrated response (Kenny et al., 2019). Nonetheless, some evidence shows that abnormal mtUPR activation in pathological conditions also has detrimental effects (Patergnani et al., 2022).

The current knowledge confirms that the compartmentalization of functions and pathways in distinct organelles is completely subdued to the intent of simplification and understanding of complex biological processes. Altered relationships between organelles often have dangerous effects and always affect overall homeostasis, usually leading to severe impairment of mitochondria. Given the fundamental role of mitochondria in cell viability, each imbalance in coordinated signaling clearly results in a threat to cell survival. Sometimes, cell death induced by protein aggregation might also be beneficial to cells, as in cancer, but the same situation is not applicable to neuronal diseases, where apoptosis leads to catastrophic outcomes.

Interestingly, these open biological questions give the chance to uncover unknown connections and mechanisms between apparently opposite pathological contexts, such as ND and cancer. Recent evidence shows that a common field might be represented by mitochondria-dependent protein aggregation and its role in inter-organelle relationships. Encouraging evidence presented in this review helps speculate that cancer might engage protein aggregation as a strategy to hinder tumor suppressor functions, such as for p53 and PTEN, whose aggregates are often resistant to lysosomal degradation. Also, Tau overexpression in gliomas ameliorates cancer outcomes by improving cell stress and consequently apoptosis. Also, amyloidogenesis, which represents a tumor suppressor checkpoint, is otherwise detrimental to neurodegeneration. Indeed, cancer cells adopt HSF1, UPR, mtUPR, and auto/mitophagy as proteotoxic stress responses to survive protein aggregation. Targeting mtUPR or HSF1 may be useful to develop therapies that increase aggregation-related stress and apoptosis in cancer while also impeding onco-suppressor aggregation. Interestingly, all these possible strategies engage a coordinated response from mitochondria, ER, and lysosomes.

The understanding of mitochondrial-related overlaps in diverse pathological situations might represent a huge turning point in the development of convergent, specific, and efficient therapeutic solutions for unmet curative needs both in cancer and ND.
